# Muscle transcriptomic investigation of late fetal development identifies candidate genes for piglet maturity

**DOI:** 10.1186/1471-2164-15-797

**Published:** 2014-09-17

**Authors:** Valentin Voillet, Magali SanCristobal, Yannick Lippi, Pascal GP Martin, Nathalie Iannuccelli, Christine Lascor, Florence Vignoles, Yvon Billon, Laurianne Canario, Laurence Liaubet

**Affiliations:** INRA, UMR1388 Génétique, Physiologie et Systèmes d’ Elevage, F-31326 Castanet-Tolosan, France; Université de Toulouse INPT ENSAT, UMR1388 Génétique, Physiologie et Systèmes d’ Elevage, F-31326 Castanet-Tolosan, France; Université de Toulouse INPT ENVT, UMR1388 Génétique, Physiologie et Systémes d’ Elevage, F-31076 Toulouse, France; INSA, Département de Génie Mathématiques, F-31077 Toulouse, France; Université de Toulouse, UMR 5219 Institut de Mathématiques, F-31077 Toulouse, France; INRA, UMR1331 ToxAlim, F-31027 Toulouse, France; INRA, UE1372 GenESI, F-17700 Surgeres, France

**Keywords:** Maturity, Survival, Birth, Muscle, Microarray, Systems biology, Pig

## Abstract

**Background:**

In pigs, the perinatal period is the most critical time for survival. Piglet maturation, which occurs at the end of gestation, leads to a state of full development after birth. Therefore, maturity is an important determinant of early survival. Skeletal muscle plays a key role in adaptation to extra-uterine life, e.g. glycogen storage and thermoregulation. In this study, we performed microarray analysis to identify the genes and biological processes involved in piglet muscle maturity. Progeny from two breeds with extreme muscle maturity phenotypes were analyzed at two time points during gestation (gestational days 90 and 110). The Large White (LW) breed is a selected breed with an increased rate of mortality at birth, whereas the Meishan (MS) breed produces piglets with extremely low mortality at birth. The impact of the parental genome was analyzed with reciprocal crossed fetuses.

**Results:**

Microarray analysis identified 12,326 differentially expressed probes for gestational age and genotype. Such a high number reflects an important transcriptomic change that occurs between 90 and 110 days of gestation. 2,000 probes, corresponding to 1,120 unique annotated genes, involved more particularly in the maturation process were further studied. Functional enrichment and graph inference studies underlined genes involved in muscular development around 90 days of gestation, and genes involved in metabolic functions, such as gluconeogenesis, around 110 days of gestation. Moreover, a difference in the expression of key genes, e.g. *PCK2*, *LDHA* or *PGK1*, was detected between MS and LW just before birth. Reciprocal crossing analysis resulted in the identification of 472 genes with an expression preferentially regulated by one parental genome. Most of these genes (366) were regulated by the paternal genome. Among these paternally regulated genes, some known imprinted genes, such as *MAGEL2* or *IGF2*, were identified and could have a key role in the maturation process.

**Conclusion:**

These results reveal the biological mechanisms that regulate muscle maturity in piglets. Maturity is also under the conflicting regulation of the parental genomes. Crucial genes, which could explain the biological differences in maturity observed between LW and MS breeds, were identified. These genes could be excellent candidates for a key role in the maturity.

**Electronic supplementary material:**

The online version of this article (doi:10.1186/1471-2164-15-797) contains supplementary material, which is available to authorized users.

## Background

Over the last decades, genetic progress has been associated with a rise in perinatal mortality in the domestic pig (*Sus scrofa*) [1]. In 2013, Strange et al. [2] noted that piglet mortality mostly occurs in the first 96 hours after birth. Because the pig is one of the most important meat-producing livestock species world-wide, this high piglet mortality at birth is a source of both economic [3] and ethical problems (public perception of the pig industry is affected by this young mortality [4]). Postnatal mortality is not an issue in pigs alone but also affects other mammals like sheep or humans [5, 6]. In humans, for example, out of 4 million cases of infant death during the first four weeks of life, 28% are due to prematurity issues [6]. Adaptation to extra-uterine life is therefore a major factor for survival.

In pigs, various factors contributing to survival at birth have already been identified. They depend on maternal traits (e.g. farrowing duration, sow health), piglet characteristics (e.g. body weight at birth, genotypes) or environment [7, 8]. As suggested by van der Lende [8], one of these factors is maturity. Maturation process was described to occur at the end of gestation, from 90 days of gestation to birth (114 days) [9]. Leenhouwers et al. [9, 10] showed that a greater physiological maturity at birth is responsible for a higher survival. Thus, a successful maturation process leads to a state of full development and promotes early survival after birth [9, 10].

Piglet maturity involves characteristics such as body size, body weight, organ characteristics and availability of body energy reserves such as glycogen or lipids [8, 9]. Maturity is also coupled with the efficiency of physiological functions like thermoregulation, which is the balance between heat loss and heat production [8, 11]. Although body weight has an influence on survival at birth, several studies suggest that it is not the only indicator of maturity [7–9, 12]. The biological background imputable to the piglet’s genetics has also been shown to impact survival [9, 10], for example, Meishan piglets have a better survival rate than Large White piglets although they are lighter at birth. Moreover, Herpin et al. [11, 13] highlighted that glucose homeostasis and body energy-glycogen storage are essential for survival.

Glycogen is the main source of polysaccharide stored in cells [11]. Glycogen storage is used to promote piglet thermoregulation at birth which takes place mainly in skeletal muscle (89% of the total glycogen) because of the absence of functional brown adipose tissue in piglets [8, 11]. After birth, glycogen levels decrease by as much as 82% in muscle to provide the energy required [14]. Proficient thermoregulation, via glycogen storage in muscle, is thus an essential prerequisite for survival after birth. Thereby, the maturation of skeletal muscle metabolism is indicative of metabolic maturity of piglet at the time of birth [11]. The muscle maturity could be defined by an immediate efficient motor function but also by an effective thermogenesis.

Some transcriptomic studies have already been performed to compare different stages of fetal muscle development in the pig. Cagnazzo et al. [15] compared seven prenatal stages (14, 21, 35, 49, 63, 77, and 91 days of gestation) in two breeds (Duroc and Pietrain) to highlight the differences in muscle development in these two breeds, while Xu et al. [16] compared a prenatal stage (65 days of gestation) with postnatal stages (3, 60 and 120 days after birth) to bring to light the mechanisms underlying muscle growth in Meishan pigs. To our knowledge, no transcriptomic studies have yet been carried out on the last phases of fetal development in connection with maturity. Here, we performed microarray analysis to describe the biological processes underlying muscle piglet maturity and identify candidate genes. The objective was to identify the genes and biological processes that are specifically involved in the differences in muscle development observed between two extreme breeds: Large White and Meishan. The Large White (LW) breed is a highly selected breed with a high rate of mortality at birth, whereas the Chinese Meishan (MS) breed produces piglets with extremely low mortality [12, 17]. The high selection in LW has led to a lower maturity of these piglets at birth [12]. MS and LW sows were inseminated with mixed semen (LW and MS). Hence each litter was composed of purebred fetuses (LW or MS) and crossbred fetuses (LWMS from MS sows and MSLW from LW sows). In the present study, we highlight key genes and biological functions involved in piglet maturity. This analysis will help to improve our knowledge of maturity in the pig.

## Results

### Power of experimental design

**Figure 1:**
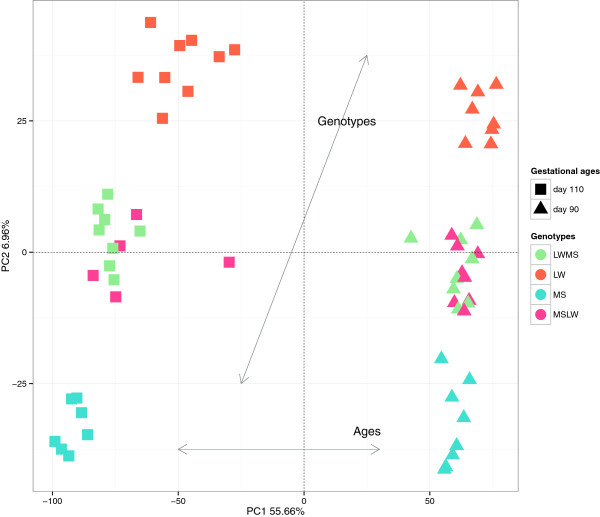
**PCA using all expressed probes.**

Microarray analysis was performed to study the last step of fetal development in the pig. Muscle samples (Longissimus dorsi) were collected from 61 fetuses in 8 different conditions (four genotypes (LW, MS, LWMS, MSLW) at two gestational ages (90 and 110 days)). After normalization, the signal intensity was found to be above background noise for 44,368 spots. Principal component analysis (PCA) was carried out to evaluate microarray quality and observe fetus dispersion (Figure 1). Principal component (PC) 1 (56% of the total dispersion) segregated fetuses according to gestational age (day 90 and day 110) while PC 2 (7%) separated fetuses according to genotype. The two groups of crossbred fetuses were mixed even with PC 3 (4%). The results of this initial descriptive study, performed without preselecting spots, showing a clear separation between gestational ages and purebreds, demonstrate that the experimental design was very powerful.

### Identification of differentially expressed genes

A mixed linear model was applied to each spot. This model involved two factors, gestational age and fetal genotype (fixed effects) as well as their interaction, and the sow as a random effect. The number of differentially expressed probes (DEP) was high even with a stringent correction for multiple tests (Bonferroni or False Discovery Rate (FDR)). Indeed, a total of 12,326 DEPs (corresponding to 5,634 unique annotated genes) were identified with a significance threshold of 1% with Bonferroni correction. This large number could be explained by a large effect of fetal gestational age and the high power of the experimental design.

The list of 12,326 DEPs was then partitioned into 4 sub-models using the Bayesian Information Criterion (BIC). Sub-model 2 (additive model for age and genotype effects) including 41% of the DEPs accounted for the largest proportion of DEPs, followed by sub-model 3 (age effect only) with 40% of the DEPs. Sub-model 4, which included the genotype effect only, contained only 3% of the DEPs. Sub-model 1 contained 2,000 DEPs (16%) (Additional file 1) and was particularly interesting because it combined the two factors of interest (gestational age and fetal genotype) and their interaction. This suggests that the last phase of the developmental process between 90 and 110 days of gestation is different across genotypes. It was therefore deemed that further analysis of this probe list would help to identify the biological processes involved in maturity.

### Ontological and functional biological analysis of differentially expressed genes

#### Biological processes enriched during the maturation process

**Figure 2:**
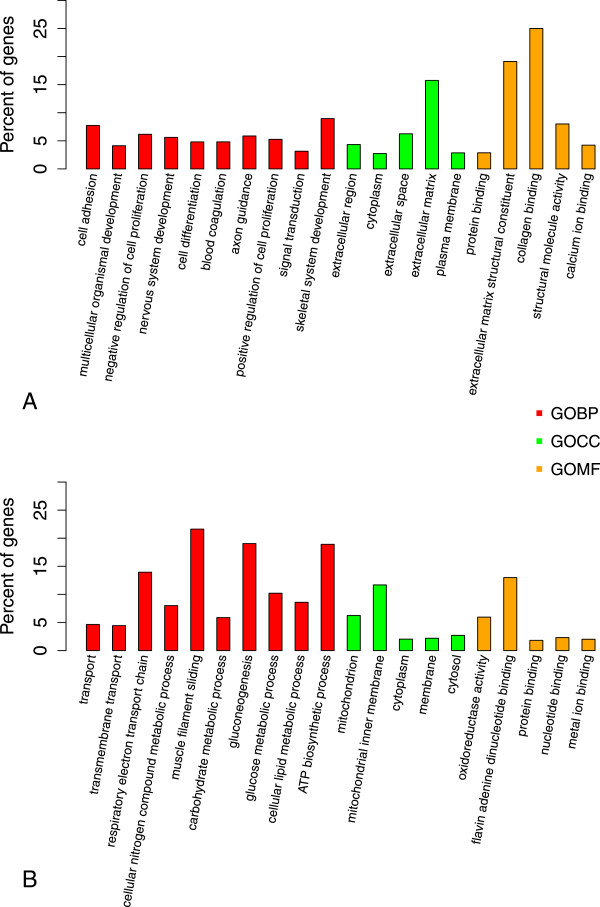
**GO analysis of annotated differentially expressed genes with a significant interaction between age and genotype.** Bar charts of the three categories of enriched GO at fetal gestational age 90 **(A)** or age 110 **(B)**: (i) the 10 first Biological Process in red, (ii) the 5 first Cellular Component in green and (iii) the 5 first Molecular Function in orange. The y axis shows the percentage of enriched genes in each category.

Gene Ontology (GO) is a standard system of classification of gene product attributes in terms of their associated biological processes, cellular components and molecular functions. Sub-model 1 identified 2,000 DEPs corresponding to 1,120 unique annotated genes. GO functional enrichment analysis was performed on two lists of genes from sub-model 1 using an absolute log2-fold change  (corresponding to an absolute fold change 1.4) between fetal gestational ages averaged over all genotypes. The first list contained 394 unique up-regulated genes at gestational day 110, and the second list contained 441 unique up-regulated genes at day 90 (Additional file 1). The top significant GO annotations indicated that the enriched biological processes at 90 days of gestation were related to muscle development (Figure 2A). Cell adhesion or signal transduction as biological processes, extracellular matrix as cellular component and extracellular matrix structural constituent as molecular function were enriched at 90 days of gestation (Figure 2A). At 110 days of gestation, the top significant enriched biological processes and molecular functions were generally involved in energy metabolism, e.g. gluconeogenesis, glucose metabolic process, cellular lipid metabolic process or oxidoreductase activity (Figure 2B). Enriched cellular components were linked, inter alia, to mitochondrion (Figure 2B). All enriched GO terms (125 GO terms at 90 days of gestation and 75 GO terms at 110 days of gestation) are presented in Additional files 2 and 3 (with unadjusted and adjusted p-values, descriptions and genes).

#### Differences of gene expression between gestational ages for each extreme fetal genotype (LW and MS)

**Figure 3:**
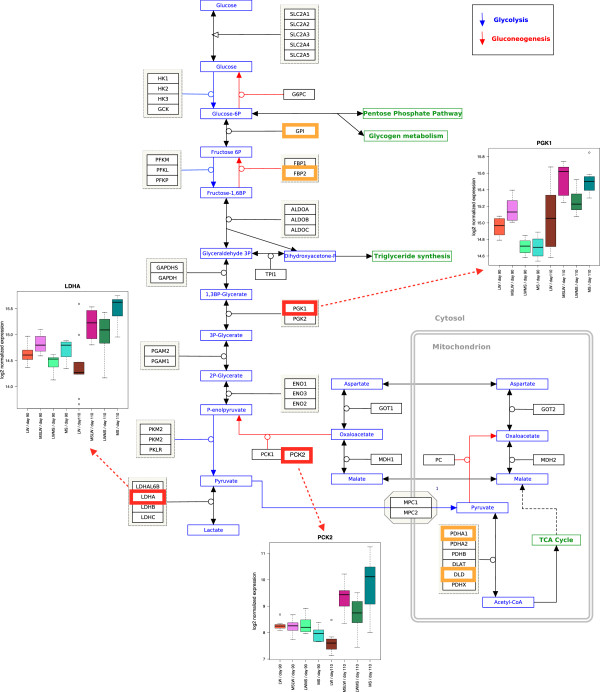
**Example of difference between LW and MS at fetal gestational age 110: pathway of Gluconeogenesis.** This figure represents the metabolic pathway of glycolysis and gluconeogenesis. Genes circled in orange (*GPI*, *GBP2*, *PDHA1*, *DLD*) are up-regulated at day 110 in KEGG pathway gluconeogenesis in both extreme breeds. Genes circled in red (*PGK1*, *PCK2*, *LDHA*) are up-regulated at day 110 in KEGG pathway gluconeogenesis in MS only. Box-plots of *PCK2*, *PGK1* and *LDHA* are added and allow to observe up-regulated genes at day 110 in MS only. The gene expression were log2 transformed. The red-circled genes illustrate the difference of maturity between MS and LW.

The aim of this part of the study was to highlight the differences of maturation process observed between purebreds (LW and MS) and more particularly to identify genes that may explain the impaired maturity of LW piglets at birth. As above, GO functional enrichment analysis was performed on the lists of genes from sub-model 1 using an absolute log2-fold change  between fetal gestational ages separately for fetuses of each breed (LW and MS). These genes were differentially expressed between the both gestational ages but not necessarily for every genotype. Interestingly, more up-regulated genes were identified in MS than in LW for several enriched biological processes, e.g various metabolic processes at 110 days of gestation or muscle development at 90 days of gestation (Table 1, Additional file 4). Another example was the glycolysis and gluconeogenesis KEGG pathway (Figure 3). Among seven enriched genes in this KEGG pathway, *PGK1* (Phosphoglycerate Kinase 1), *PCK2* (Phosphoenolpyruvate Carboxykinase 2, mitochondrial also known as *PEPCK*) or *LDHA* (Lactate dehydrogenase A) were only up-regulated in MS at day 110 (see box-plots on Figure 3). These genes may illustrate the differences in the muscle maturation process between purebred MS and LW piglets.

**Table 1:** **Table of six enriched GOBP at day 90 or 110 in the two extreme breeds**

Day	Items	GOBP Terms	Genes
90	GO:0007275	Multicellular organismal development	*TCF12* *MGP* *KLF3* *FRZB* *DIAPH2* *IGF2* *SEMA4D* *GPSM1* *MESP1* *CCBE1* *VEGFC* *CREM* *RYBP* *JAG1* *KDR* *CSPG4* **CECR1**
	GO:0030154	Cell differentiation	*TCF12* *MGP* *FRZB* *DIAPH2* *SEMA4D* *GPSM1* *VEGFC* *CREM* *CSPG4* **SH2B3**
	GO:0001501	Skeletal system development	*FRZB* *IGF2* *GDF11* *IGF1*
110	GO:0006094	Gluconeogenesis	*PCK2* *GPD1* *PGK1*
	GO:0006006	Glucose metabolic process	*PCK2* *UPG2* *PGK1* *PYGL* *SORD*
	GO:0044255	Cellular lipid metabolic process	*GPD1* *OXCT1* *SLC25A20*

In italic, genes are up-regulated in MS only, and in bold, genes are up-regulated in LW only. Genes up-regulated in MS and LW are not represented. The complete list of genes up-regulated in MS and/or LW is given in Additional file 4.

#### Differences between extreme fetal genotypes (LW and MS) with a relevance network approach

**Figure 4:**
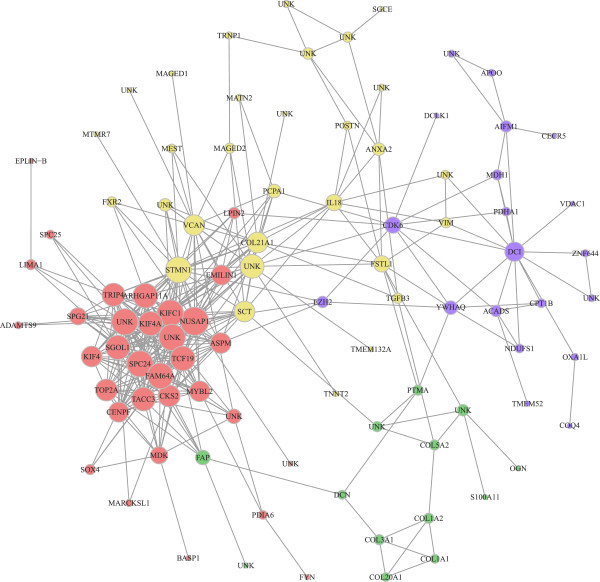
**Largest connected component of the relevance network.** Each node in the graph represents a gene and each edge corresponds to a Pearson correlation between two genes above the defined threshold (|*r*|>0.98). The size of each node is proportional to its degree. The graph included 96 nodes and 381 edges. Modularity was maximized to find communities in the graph. Four communities were found and labelled with color (red for community 1, yellow for community 2, green for community 3 and blue for community 4). The percentages represent the number of genes in each community. For each community, we studied biological processes with GO functional enrichment analysis. Communities 1, 2 and 3 were mainly involved in muscle development, such as cell division, cell adhesion and collagen. Community 4 was mainly involved in metabolism like the fatty acid one. All enriched GO Terms are detailed in the. UNK stands for 'unknown’.

**Figure 5:**
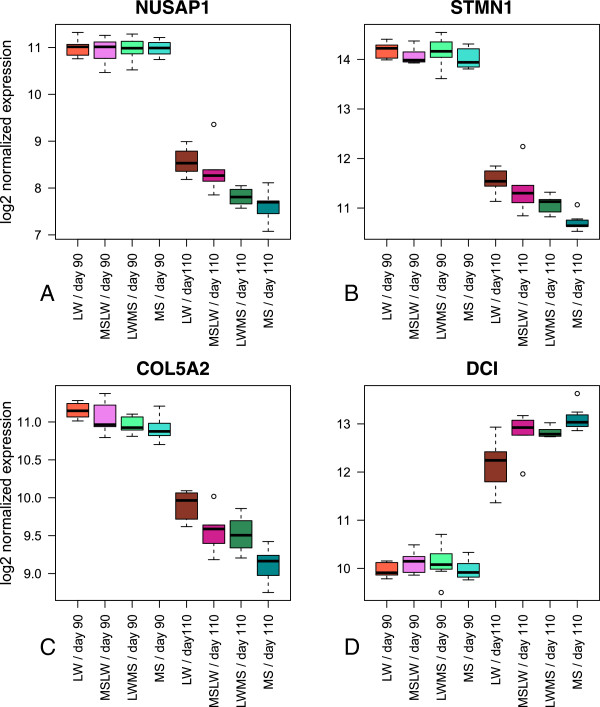
**Box-plot representation of gene expression with high degree in each community.** The gene expression were log2 transformed. **(A)** *NUSAP1* in community 1. **(B)** *STMN1* in community 2. **(C)** *COL5A2* in community 3. **(D)** *DCI* in community 4.

In contrast with bibliometric networks (e.g. with Ingenuity), a relevance network approach can use the transcriptomic information of both annotated and unannotated genes. Influencial genes can be found by looking at degrees and betweeness centrality. The degree is the number of edges a gene, as a node, has to other genes. A gene with the highest degree is usually considered as a hub. Betweenness centrality quantifies the number of times a gene acts as a bridge along the shortest path between two other genes. A relevance network between the 1,516 unique genes (annotated or not) from sub-model 1 was built on Pearson correlation *r* using a threshold of |*r*|>0.98. It should be noted that with a lower threshold the resulting graph was too large and too highly connected to be interpreted (Additional file 5). The largest connected component obtained was composed of 96 nodes and 381 edges (Figure 4, Additional file 6). *NUSAP1* (Nucleolar and spindle associated protein 1) was the gene with the highest degree, while *CDK6* (Cyclin-dependent kinase 6) was the gene with the highest betweenness centrality (Additional file 6). By maximizing the modularity criterion, four communities were identified in this graph (Figure 4). According to functional enrichment analysis, each community was related to a particular biological function: (*i*) community 1 was involved in cell division and nucleus, (*ii*) community 2 in cell adhesion and extracellular matrix, (*iii*) community 3 in collagen, while (*iv*) community 4 was involved in regulation of the fatty acid metabolism and oxidation-reduction process. Detailed results (unadjusted and adjusted p-values, descriptions and genes) of enriched GO analysis are presented in Additional file 7. Genes belonging to communities 1, 2 and 3, e.g. *NUSAP1*, *STMN1* (Stathmin 1) and *COL5A2* (Collagen alpha-2(V) chain), were mainly up-regulated at day 90, with a higher expression in LW than in MS at day 110 (Figure 5). On the contrary, the genes of community 4, e.g. *DCI* (or *ECI1*) (Enoyl-CoA Delta Isomerase 1), were mainly up-regulated at day 110 with a higher expression in MS (Figure 5). The expression profiles of the relevance network highlighted a delay of gene expression in LW fetuses at 110 days of gestation.

From a methodological point of view, it should be noted that a bias could have been introduced by the choice of the Pearson correlation to represent the relationships between genes [18]. Partial correlation, which discriminates between direct and indirect relationships, may have led to more relevant measurements of the direct dependence between variables [18, 19]. However, in our study, the genes of interest were too numerous (1,516 unique genes) and too highly correlated to compute partial correlations correctly.

Taken together, the up- or down-regulation of the genes involved in these four communities was delayed at 110 days of gestation in LW fetuses compared with MS fetuses. These results are consistent with a lower maturity (and a higher mortality) of LW piglets at birth compared to MS piglets Results section and Additional file 7.

### Influence of the paternal or maternal genome on gene expression

This experiment used a reciprocal design to independently evaluate the effect of each parental genome on the maturation process (MS and LW). In other words, if both genomes contributed to the same extent, as expected in the Mendelian context, gene expression would be identical in the two sets of crossbred fetuses. However, the reality is that some genes are not regulated in the same manner depending on the origin of the allele. Eight hundred and five probes were identified to be impacted by the parental genotype in interaction with gestational age (FDR < 1%). One hundred and six unique annotated genes (164 probes identified) were influenced by the maternal genotype and 366 unique annotated genes (641 probes identified) were influenced by the paternal genotype (Additional files 8 and 9). It should be noted that 19 probes (12% of the 164) influenced by the maternal genome were located on chromosome X versus 19 probes (3% of the 641) influenced by the paternal genome. Because only male fetuses were studied, all genes from the X chromosome were of maternal origin. Moreover, 4 probes out of the 164 probes influenced by the maternal genome were located on mitochondrial chromosome.

**Figure 6:**
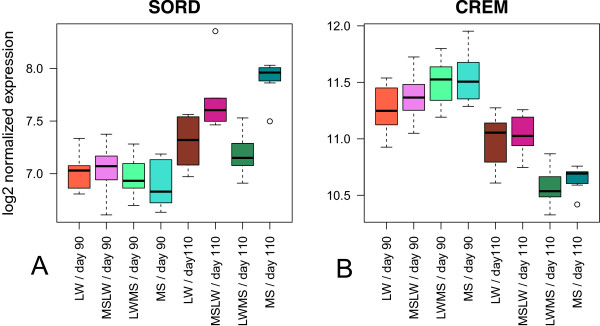
**Box-plot representation of gene expression of** ***SORD*** **and** ***CREM*** **.** The gene expression were log2 transformed. **(A)** *SORD* with an impact of the paternal genotype at day 110. **(B)** *CREM* with an impact of the maternal genotype at day 110.

Several previously identified genes (of sub-model 1) were also influenced by a parental effect (602 probes). For example, *PCK2* and *LDHA* were impacted by the paternal genome as can be seen in the box-plots (Figure 3). Other identified genes, such as *SORD* (sorbitol dehydrogenase) and *CREM* (cAMP responsive element modulator), showed both a parental effect and a difference between purebred fetuses (Table 1, Figure 6). *CREM* expression was influenced by the maternal genotype, whereas *SORD* expression was influenced by the paternal genotype (Figure 6). These genes illustrate the possible impact of the parental genotype on gene expression, and its effect on maturity.

### Validation of differential expression by quantitative real time PCR

To validate the microarray results, the expression profiles of 10 genes of interest were monitored using qRT-PCR. The 10 selected genes showed differential expression for the two fixed effects and their interaction in the microarray (sub-model 1). The similarity between the results obtained with the microarray and qRT-PCR confirmed the accuracy of gene expression measurements (illustrated in Additional file 10). Indeed, the Pearson correlation between the differences in expression measured by qRT-PCR and microarray was greater than 0.70 for all genes, except *IL1RAPL2* and *SPG7* (Table 2). The high variability obtained by qPCR suggests that all genes were not highly correlated, especially in the LW sample at 110 days of gestation. Nevertheless, the correlation obtained confirms our previous results and all the expression profiles are similar.

**Table 2:** **Correlation between qPCR expression with microarray expression for selected genes (n = 43)**

Genes	Pearson’s correlation	P-value
*ARG2*	0.95	<0.001
*PHKA1*	0.86	< 0.001
*SLC38A4*	0.84	<0.001
*DLK1*	0.83	< 0.001
*RASGRP3*	0.81	< 0.001
*GPD1*	0.76	< 0.001
*DUT*	0.74	< 0.001
*GBP1*	0.71	< 0.001
*IL1RAPL2*	0.65	< 0.001
*SPG7*	0.40	0.008

## Discussion

### Important transcriptomic changes between 90 and 110 days of gestation

The statistics chosen here to detect DEPs made no use of a minimum fold change filter. This choice was based on the complexity of the experimental design (8 conditions in a 2 by 4 factorial design) making a fold change calculation difficult. Moreover, no associated values indicate the level of confidence in the designation of genes as differentially expressed or not differentially expressed with such a fold change filter. A fold change filter was applied only at the point of gene function enrichment. The counterpart of this choice may be a potential for over-interpretation of data, by extracting genes with very small changes in expression. With a commonly-used threshold in FDR of 1*%*, as high as 28,833 probes were found DE, corresponding to 65*%* of expressed probes of the microarray. We chose instead a conservative cutoff of 1*%* with a Bonferroni correction for multiple testing. A high number of DEPs (12,326 probes corresponding to 28% of the expressed probes) was obtained even with this stringent correction. This high number demonstrates, as does PCA (Figure 1), that the choice of breeds and the two gestational ages were highly relevant in order to study contrasted situations linked to the maturation process. The important impact of genotype was expected in agreement with Hazard et al. [20], where 82% of the differentially expressed genes were impacted by the genotype, comparing LW and MS in another experimental context.

Among these DEPs, 11,952 probes (97%) were influenced by the gestational age of fetus (genes in sub-models 1, 2 and 3). PCA of all expressed genes also showed the prime importance of fetal gestational age (see the first axis on Figure 1). This high number of DEPs related to gestational age, about 27% of the probes in the microarray, could be explained by a switch of gene expression between 90 days and 110 days of gestation. Muscle development is described to end around 90 days of gestation [21], and gives way to the maturation process in order for the organs and tissues to be functional at birth. In other words, for the fetus to be able to adapt to the extra-uterine environment, fetal tissues must have acquired complete functionality at birth.

Using embryo transfers between two breeds (MS and Yorkshire), of the results published by Wilson et al. [22] and Biensen et al. [23, 24] suggested that fetal development is determined by the uterine environment until 90 days of gestation, regardless of the fetal genotype. After 90 days of gestation, the last phase of fetal development is preferentially modulated by the fetal genotype with mechanisms specific to each genotype [23]. Our experiment explored this final step of development in utero. We revealed the importance of the transition between fetal development and metabolism in muscle tissue for survival (i.e. energy storage and function: gluconeogenesis, glycolysis and fatty acid metabolisms).

### Main biological mechanisms of maturity in pigs

To identify the biological processes underlying muscular maturity, functional enrichment analysis was performed on two gene lists from sub-model 1 (model which combined two factors, gestational age and fetal genotype (fixed effects), as well as their interaction, and the sow as random effect). The first list consisted of 441 up-regulated genes at day 90, and the second consisted of 394 up-regulated genes at day 110. These genes were chosen because of their interaction between the gestational age and the fetal genotype. We wanted to observe differences in the muscle maturation process between the both extreme breeds.

Enriched biological functions at 90 days of gestation were involved in muscle development and reflected processes such as cell adhesion, signal transduction or skeletal muscle development (Figure 2A). These results are consistent with the second phase of muscular development known to occur in pigs between 55 and 90 days of gestation [21, 25]. In the pig, ontogenesis of muscle fibers is a biphasic phenomenon [21, 25, 26]. A first generation of myofibers develops between 35 and 55 days of gestation, followed by a second generation between 55 and 90 days of gestation. The second generation develops around each primary myotube using it as a scaffold and determines the number of myofibers [21, 25]. The total number of myofibers is definitively fixed at approximately 90 days of gestation [27]. Moreover, it has been reported that the number of myofibers is lower in MS than LW which may explain the small postnatal muscle growth capacity of MS pigs, in particular of large glycolytic muscle, e.g. longissimus muscle [27].

**Figure 7:**
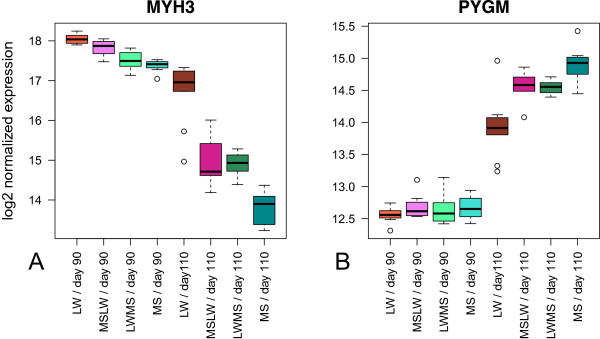
**Box-plot representation of gene expression of (A)** ***MYH3*** **and (B)** ***PYGM*** **.** The gene expression were log2 transformed.

Among genes of sub-model 1 (Additional file 1, Figure 7A), the embryonic heavy chain isoform of myosin (corresponding to *MYH3* gene) is described to be only expressed between 50 days of gestation and birth [28]. In this study, while *MYH3* expression decreased in MS just before birth, its expression in LW stayed at a high level even at 110 days of gestation. It may correspond to the delay of variation of expression mainly observed between LW and MS at 110 days of gestation, suggesting a possible state of immaturity at birth as expected in LW. In another way, a variation of copy number of *MYH3* has been recently identified in cattle, that was positively correlated with its transcript expression and body traits (body height, body weight and body size) [29]. It would be possible to imagine that selection on growth traits impacted polymorphisms in this gene, such as CNV (Copy number variations). In our study, *MYH3* expression is eight fold higher in LW than in MS just before birth. Further studies would be needed to explore the relationship between this possible delay of maturity in LW with the differential proportion of glycolitic myofiber observed between LW and MS (larger proportion in LW [27]). It was observed that comparison between LW and MS may suggest that intensive selection for lean muscle growth induced a shift in muscle metabolism toward a more glycolytic and less oxidative myofiber type [27]. However, it is not clear if a functional link may be proposed to explain an effect of the genetic difference of a higher expression of *MYH3* at birth with the metabolic status in longissimus dorsi muscle. In another way, the expression of *PYGM* gene (glycogen phosphorylase) was up-regulated at 110 days of gestation with a higher expression in MS (Figure 7B) meaning a delayed expression in LW. A higher activity of the glycogen phosphorylase at protein level would illustrate a higher capacity to degrade the glycogen store at birth to produce energy.

The pattern of muscle development was confirmed by the results of our relevance network in which the genes involved in development were up-regulated at 90 days of gestation. For example, *NUSAP1*, the gene with the highest number of connections to other nodes (or degree) in our network, is a key gene for spindle microtubule organization and has been identified as playing a central role in regulating mitosis depending on its phosphorylation state [30, 31]. The gene with the highest degree of relevance in community 2, *STMN1*, is also involved in the regulation of the microtubule filament system [32]. Moreover, part of the collagen family was present and connected in community 3 (Figure 4). *CDK6* is an important gene for two reasons: it has the highest betweeness centrality, indicative of its prime role in the structure of the network, and it links community 4 (muscle metabolism, with genes up-regulated at 110 days) with the remaining three communities (involved in muscle development, genes up-regulated at 90 days). Interestingly, *CDK6* is up-regulated at 90 days, unlike genes of community 4. This member of the cyclin-dependent protein kinase family regulates cell cycle progression and is involved in the regulation of skeletal muscle regeneration [33, 34]. Because these genes are down-regulated between 90 and 110 days of gestation, our results are in accordance with the previous hypothesis that muscle development “switches off” at around 90 days.

At 110 days of gestation, the enriched biological functions detected were involved in energy metabolism, especially in gluconeogenesis and cellular lipid processes (Figure 2B). In contrast with intra-uterine life where fetal body temperature depends on the sow, autonomous thermoregulation must occur immediately upon birth for the piglet to survive [11]. Energy reserves, i.e. glycogen and fat, must therefore be maximal in the neonatal period because the piglet cannot oxidize protein efficiently before 5-7 days of life [11]. In pig fetuses, glycogen is stored in skeletal muscle (89% of all glycogen reserves [11]) and liver, because pigs lack brown adipose tissue [8, 35]. The initial role of muscle glycogen, in addition to motor function, is postnatal thermogenesis, especially prior to colostrum intake. Later, if energy intake is insufficient, the piglet draws down on its muscle glycogen reserves [11]. Large amounts of glycogen are therefore stored in muscle before birth (around 114 days of gestation) [11]. The genes of community 4 that were up-regulated at 110 days of gestation were involved in fatty acid metabolism which is also important for forming the body energy reserves required at birth [11]. *DCI*, which was the gene with the highest degree of relevance in this community and the second betweenness centrality in our network, encodes a key mitochondrial enzyme involved in beta-oxidation of unsaturated fatty acids [36].

Globally, the process of muscle maturation was the same in each studied genotype: muscle fiber proliferation is switched off at around day 90, and the enzymes coding genes for glycogen and lipid metabolism are up-regulated at around day 110 to ensure regulation of mechanisms essential for survival at birth. This is also in line with the changes in gene expression that have been reported at the end of gestation.

### Contrasted maturation process between extreme breeds

Even if the overall muscle maturation process is the same for each genotype, some important differences were found between the extreme breeds that affected crucial biological processes (Table 1 and Additional file 4). Genes such as *PGK1*, *PCK2* or *LDHA*, encoding key enzymes involved in gluconeogenesis and glycolysis KEGG pathway, were up-regulated at day 110 in MS only (Figure 3). It should be noted that *PCK2* and *LDHA* were down-regulated at day 110 compared to day 90 in LW (see box-plots on Figure 3). Of special interest is *PCK2* that encodes an enzyme that catalyzes the irreversible conversion of oxaloacetate (OAA) to phosphoenolpyruvate (PEP), the rate-limiting step in the metabolic pathway that produces glucose from lactate and other precursors derived from the citric acid cycle [37]. This result may be surprising as gluconeogenesis is more often described to be a liver function than a property of muscle [38].

Body energy reserves, i.e. glycogen and fat, are important predisposing factors involved in the maturation process [8, 9]. The fact that a piglet is unable to produce heat, may be the result of an immature metabolic capacity. A weaker expression of the genes (e.g. *PCK2*, *PGK1* or *LDHA*) involved in these metabolic pathways between the two gestational ages may cause the lower maturity observed in LW, and therefore be responsible for the larger proportion of deaths at birth in this breed. Moreover, muscle glycogen content has been already studied just before birth [9, 10]. The animal’s requirement for energy is maximum in the neonatal period to promote thermoregulation and growth [11]. It strongly suggested that piglets with high value for survival, like MS, have a higher ability to maintain glucose levels during and after farrowing and are better able to maintain body temperature. For a long time, the storage and mobilization of the glycogen in muscle was known to be essential for survival at birth [39].

Genetic selection has been shown to alter genes which may be associated with marked differences in maturity between MS and LW [40]. Canario et al. [12] have already shown that selection for leanness in LW resulted in a lower maturity of piglets at birth, for example, with an effect on the body protein content and liver glycogen stores. Genetic polymorphisms could affect the genes involved in these processes. For example, *PGK1* showed a greater variability of expression in LW suggesting a possible underlying polymorphism in purebred LW fetuses (see box-plot on Figure 3). *PCK2* and *LDHA* showed distinct expression profiles in MS and LW (down-regulated at day 110 compared to day 90 in LW) (see box-plot on Figure 3).

In addition to the genes up-regulated at 110 days of gestation in MS pigs only, the lesser maturity of LW piglets could also be explained by a delay in gene expression at the end of the intra-uterine developmental period. As found with the relevance network approach (Figure 4), most genes in the communities involved in development were down-regulated just before birth, such as *NUSAP1*, *STMN1* or *COL5A2*. These genes had a higher expression in LW than in MS (Figure 5). Most genes of community 4 (metabolic processes), such as *DCI*, were up-regulated at 110 days of gestation and the related genes were mainly expressed at a higher level in MS than in LW. Our relevance network approach is therefore in accordance with the assumption that MS piglets are more mature than LW piglets. Furthermore, the network may also provide information on unannotated genes by guilt-by-association. These results suggest that the genetic selection may affect genes involved in the muscle metabolic capacity confirming the initial assumption that MS newborns are more mature than the LW ones [17] and enables us to suggest candidate genes for piglet maturity.

### Impact of the parental genotypes

We identified a great number of annotated genes (472) that were impacted by one of the two parental genotypes during maturation process: 106 genes were influenced by the maternal genotype and 366 genes by the paternal genotype (Figure 6 and Additional files 8 and 9). The influence of the parental genotype was found for several genes, such as *CREM* and *SORD* (Figure 6), which also showed difference between extreme breeds (Table 1). Some of these genes are known to be related to imprinting in pigs. Many studies of genome scanning for QTL (Quantitative Trait Loci) in pigs revealed that many of them are maternally or paternally imprinted, which significantly affect growth, backfat thickness, carcass composition and reproduction [41]. Among these genes, we identified *IGF2* (Insulin-like growth factor 2) [42] and *MAGEL2* (MAGE-like 2) [43]. The selection pressure for enhancing lean meat content was described to be related to increase *IGF2* transcript expression in muscle [44]. The imprinting of the *MAGEL2* gene is highly conserved among species [43]. These results are consistent with the theory explaining parental conflict during gestation [45]. The parental genomes have a direct impact on fetal gene expression (2.8% of expressed genes in our microarray). The genetic component of piglet survival consists of a maternal genetic component (genotype of the mother) and a direct genetic component (genotype of the piglet) [9]. In this context, at the end of gestation, the paternal expression genes are up-regulated to allow the fetus to express its growth potential [23]. It has already been shown that paternally expressed genes are not essential for the initiation of fetal development [46], but their role becomes more critical at the end of gestation.

Moreover, these genes could play a key role in the maturation process. A large number of imprinted genes in humans are known to affect metabolic parameters such as glycogen metabolism [47]. For example, the expression of *MAGEL2*, known to have an effect on metabolic parameters and fetal growth [47], is impacted by the paternal genotype in our study (Additional file 9). This gene was up-regulated at 90 days of gestation and further up-regulated in paternal genotype MS. The MS paternal genotype therefore represents a strong genetic component for this gene’s expression. Thus, the delay in the maturity of LW piglets may also be explained by differential impacts of the parental imprinting of some genes involved in metabolic processes. However, in our study, the relative involvement of each parental genome was studied only by comparison between the reciprocal crossed fetuses then no affirmation could be done on the imprinting status of the identified genes. Allele specific expression could be investigated in the future.

## Conclusions

Our experimental design was very powerful in order to unravel the biological processes underlying the last phase of muscle development, and identify key muscle differences between LW and MS pigs that could explain lesser maturity of LW piglets at birth. Biological functions and genes involved in maturity have been identified in each breed. This study shows that a considerable transcriptomic change occurs between 90 and 110 days of gestation, and corresponds to a switch between muscle development and muscle metabolism. This study also highlighted genes with differences of expression between extreme breeds LW and MS, such as *PCK2*, *PGK1* and *LDHA*. These genes play roles in the implementation of the muscle metabolic processes necessary for thermoregulation at birth. These processes are also under the conflicting regulation of the two parental genomes with a predominance of the paternal genotype which affects genes such as *MAGEL2* and *IGF2*. These results are a first step in understanding the global system biology of piglet maturity. Some genes described in this report could be candidates to explore the genetic control of maturity. Futher functional and genetical studies may be focused on the LW breed with its increased mortality at birth, and then be continued to identify the genetic mechanisms underlying the differences in maturity.

## Methods

### Ethical statement

All animal use was performed under European Union legislation (directive 86/609/EEC) and French legislation of région Midi-Pyrénées in France (Décret n ∘:2001-464 29/05/01; http://ethique.ipbs.fr/sdv/charteexpeanimale.pdf; accreditation for animal housing number C-35-275-32). The technical and scientific staff obtained individual accreditation (Ref: MP/01/01/01/11) from the ethics committee (région Midi-Pyrénées - France; http://comethmp.ipbs.fr/) to experiment on living animals. All pigs used in this study were males and were obtained by caesarean.

### Experimental design and RNA preparation

To assay for changes in gene expression during piglet maturity, mRNA was isolated from 64 fetal muscle samples (longissimus dorsi) in 8 different conditions: two fetal gestational ages (day 90 and day 110) associated with four fetal genotypes. The four fetal genotypes consisted of two extreme breeds for mortality at birth (LW and MS) and two crosses (MSLW and LWMS). MS and LW sows were inseminated with mixed semen (LW and MS) so that each litter was composed of purebred fetuses (LW or MS) and crossbred fetuses (LWMS from MS sows and MSLW from LW sows). Total RNA was isolated from each of the 64 muscle samples. Briefly, muscle samples were disrupted, homogenized and ground to a fine powder by rapid agitation for 1 min in a liquid-nitrogen cooled grinder with stainless steel beads. An aliquot of 100 mg of the fine powder was then processed for total RNA isolation and purification using Trizol (Invitrogen, France) and the Nucleospin RNA II kit (Macherey-Nagel, France) according to the manufacturer’s instructions. The method included a DNase digestion step to remove contaminating DNA. The extracted total RNA was eluted in 300 *μl* of RNase-free water and stored at -80°C. RNA quality and concentration were verified using an Agilent 2100 bioanalyzer (RNA solutions and RNA 6000 Nano Lab- Chip Kit, Agilent Technologies France, Massy, France).

### Microarray description

The microarray GPL16524 (Agilent technology, 8 × 60K) used in this experiment consisted in 43,603 spots derived from the 44K (V2:026440 design) Agilent porcine specific microarray, 9,532 genes from adipose tissue, 3,776 genes from the immune system and 3,768 genes from skeletal muscle (Liaubet et al. (personal communication), unpublished data).

After quality control and a quantile normalization step as described in [48], the data of fluorescence signal from 61 microarrays containing 44,368 spots were kept for further analysis and log2 transformed. These spots correspond to 34,945 annotated genes, i.e. 16,712 unique annotated genes. It is important to consider that the annotations are constantly being improved due to annotation issues in pig. However, all cited genes were checked. Raw data and information are available in NCBI (GEO accession number GSE56301).

### Statistical analysis

Statistical analyzes were performed with R 3.0.2 software [49]. To analyze jointly differences between breeds and gestational ages, the following mixed linear model was fitted to each probe (R nlme package, lme function [50]):
1

with *i*∈{*d*90,*d*110}, *j*∈{*LW*,*MS*,*LWMS*,*MSLW*}, *k*=1,…18,  independent and identically distributed (iid) and  iid. *S*_*k*_ and *ε*_*ijk*_ are mutually independent. *y*_*ijk*_ is the expression of the probe (gene) being studied, *μ* a general mean of the considered gene expression and *ε*_*ijk*_ is a residual. This model includes two fixed effects and their interaction: *A*_*i*_ is the effect of fetal gestational age *i*, *FG*_*j*_ the effect of fetal genotype *j* and *A*.*FG*_*ij*_ the interaction effect between gestational age *i* and genotype *j*. *S*_*k*_ represents the random sow effect.

To identify differentially expressed probes (DEPs) for gestational age and/or genotype, the mixed linear model (1) was fitted to the microarray data. A F-type test was performed by comparing the complete model (1) and the reduced model *y*_*ijk*_=*μ*+*S*_*k*_+*ε*_*ijk*_. A correction for multiple testing was then implemented using Bonferroni [51] or False Discovery Rate (FDR) [51, 52] using the multtest R package [53].

The list of DEPs was then partitioned into 4 sub-models. Sub-model 1 combined the two fixed effects and their interaction. Sub-model 2 involved the two fixed effects in an additive manner. Sub-model 3 included only the fetal gestational age effect whereas sub-model 4 included only the fetal genotype effect. All models included the random sow effect. In summary:
2

The Bayesian Information Criterion (BIC) was used to associate each DEP with one of these four sub-models.

To analyze the parental impact, the following mixed linear models involving the two parental genotypes were fitted to each probe:
3

with *i*∈{*d*90,*d*110}, *j* and *k*∈{*LW*,*MS*,*LWMS*,*MSLW*}, *l*=1,…18,  independent and identically distributed (iid) and  iid. *S*_*l*_ and *ε*_*ijkl*_ are mutually independent. *y*_*ijkl*_ is the expression of the probe (gene) being studied, *μ* a general mean of the considered gene expression and *ε*_*ijkl*_ is a residual. This model includes two fixed effects and their interaction: *A*_*i*_ is the effect of fetal gestational age *i*. *MG*_*j*_ is the effect of maternal genotype *j* and *A*.*MG*_*ij*_ the interaction effect between gestational age *i* and maternal genotype *j*. *PG*_*k*_ is the effect of paternal genotype *k* and *A*.*PG*_*ik*_ the interaction effect between gestational age *i* and paternal genotype *k*. *MG*.*PG*_*jk*_ is the interaction between parental genotypes.

To identify DEPs, these mixed linear models were fitted to the microarray data. F-type tests were performed by comparing the complete model (3) and two reduced models: one without the interaction between gestational ages and maternal genotype to identify genes influenced by the maternal genotype and the other without the interaction between gestational ages and paternal genotype to identify genes influenced by the paternal genotype. A correction for multiple tests was then implemented using Bonferroni [51] or FDR [51, 52] using the multtest R package [53] as previously.

### Gene Ontology functional enrichment analysis

Functional annotation of genes from sub-model 1 based on Gene Ontology (GO) was provided by GeneCoDis 3.0 software [54]. Enrichment analysis was applied to lists of genes selected for an absolute log2-fold change greater than  between both gestational ages. This threshold of a -log2-fold change was used to obtain two values: up-regulated genes at day 90 or up-regulated genes at day 110. This threshold was applied to ensure that only the genes with a minimal change between gestational ages were retained for the GO functional enrichment analysis. The two lists contained up-regulated genes at 90 days and up-regulated at 110 days respectively. To set the statistical enrichment of a particular biological function, a hypergeometric test was used. Resulting p-values were adjusted for multiple tests using the FDR approach (FDR < 1%).

### Relevance network

A relevance network is a graphical model displaying genes as edges and relationships (correlations here) between genes as vertexes [55]. In our study, a network building pipeline, using the igraph R package [56], was decomposed into three steps. In a first step, a similarity matrix *S* was calculated using the Pearson correlation coefficient between pairs of genes. In a second step, *S* was transformed into a binary adjacency matrix *A* using hard thresholding. This matrix *A* was composed of 0 and 1 depending on whether the correlation coefficient was lower or greater than 0.98 (in absolute value), respectively. The final step consisted in a graph representation of *A*. An edge was present between two nodes (genes) *i* and *j* if the value *a*_*ij*_ in *A* was 1.

To determine communities in the graph, a fast-greedy algorithm was used to optimize the modularity of a partition of the network [57]. The modularity is a measure of the quality of communities in the network: highly connected genes within each community, and lowly connected genes between communities. Finally, GO functional enrichment analysis was performed to determine enriched biological processes in each community. In addition to dividing the network structure into sub-networks, influential genes were highlighted as described in Villa et al. [19] based on other criteria, i.e. degree and betweenness centrality.

### Quantitative real time RT-PCR analysis for gene expression

Gene primers were designed from pig genes taking into account intron-exon organization using Primer3 software (http://frodo.wi.mit.edu/primer3/). Sequences are available in Additional file 11. RNA samples were reverse transcribed from 1 *μg* as previously described in [58]. The resulting cDNA samples were completed to 50 *μl*. The assay for each gene consisted of four replicates per genotype and development stage (from the 61 used in the microarray experiment) and negative controls.

The expression of 10 genes was analyzed using 48.48 Dynamic Array™ IFCs and the BioMark™ HD System from Fluidigm. Two specific target amplifications (STA) were performed on cDNA muscle samples according to the manufacturer’s recommendations. As previously described [59], a 14 cycle STA treated with Exonuclease I was performed, diluted and transferred to the BioMark™ HD for final STA. The efficiency of PCR amplification was determined specifically for each gene, by serially diluting (1, 1:2; 1:2; 1:2) the muscle cDNA pool.

Data was then analyzed using Fluidigm Digital PCR Analysis software with the Linear (Derivative) Baseline Correction Method.

After determination of the threshold cycle (Ct), the Pfaffl method [60] was applied as described in [59] to calculate the relative expression of each gene. *HPRT*, which was not regulated during the maturation process, was used as the reference gene. Pearson’s correlations were calculated between microarray expression and qPCR values.

## Availability of supporting data

Microarray data are MIAME compliant and available in Gene Expression Omnibus (GEO, http://www.ncbi.nlm.nih.gov/geo/) through the accession number GSE56301.

## Electronic supplementary material

Additional file 1: **Complete list of differentially expressed probes of sub-model 1 '.xlsx’ file.** Features of 2000 genes of sub-model 1 (Gene symbol, gene name, probe name, p-value and log2-fold change (d110/d90)). P-value, Bonferroni and FDR were obtained by a F-type test comparing the complete model (1) and the reduced model *y*=*μ*+*S*+*ε*. (XLSX 228 KB)

Additional file 2: **Complete list of enriched GO (Biological Process (BP), Molecular Function (MF) and Cellular Component (CC)) at day 90 '.xlsx’ file.** The file gives the GO items, the corresponding functions, the classes of ontology, the lists of genes, the numbers of genes in the input lists and the reference lists and p-values (unadjusted and FDR). (XLSX 30 KB)

Additional file 3: **Complete list of enriched GO (BP, MF and CC) at day 110 '.xlsx’ file.** The file gives the GO items, the corresponding functions, the class of ontology, the list of genes, the number of genes in the input list and the reference list and p-values (unadjusted and FDR). (XLSX 23 KB)

Additional file 4: **Complete list of the first twelve enriched GOBP at day 90 or at day 110 in LW or MS '.xlsx’ file.** The first twelve enriched GOBP in LW and MS at day 90 and day 110 (Items, functions, gene lists and p-value (unadjusted and FDR)). In red, genes are up-regulated in MS only and in blue, genes are up-regulated in LW only. In black, genes are up-regulated in LW and MS. (XLSX 19 KB)

Additional file 5: **Frequency distribution of Pearson’s correlation network '.pdf’ file.** Frequency distribution of Pearson’s correlation between the entire set of 1516 genes (annotated or not) used to build our network. (PDF 36 KB)

Additional file 6: **Features of genes in the relevance network '.xlsx’ file.** Gene names, probe name, degree, betweenness centrality and community of genes in the relevance network. (XLSX 17 KB)

Additional file 7: **Complete list of enriched GO (BP, MF and CC) in the four communities of the relevance network '.xlsx’ file.** The file gives the GO items, the corresponding functions, the classes of ontology, the lists of genes, the numbers of genes in the input lists and the reference lists and p-values (unadjusted or FDR). (XLSX 21 KB)

Additional file 8: **Complete list of differentially expressed probes impacted by maternal genome '.xlsx’ file.** Features of 164 DEPs using model (3) (Gene symbol, gene name, probe name, p-value and *Sus scrofa* chromosome localization). P-value, Bonferroni and FDR were obtained by a F-type test comparing the complete model (3) and the reduced model without interaction between gestational age and maternal genotype. (XLSX 37 KB)

Additional file 9: **Complete list of differentially expressed probes impacted by paternal genome '.xlsx’ file.** Features of 641 DEPs using model (3) (Gene symbol, gene name, probe name, p-value and *Sus scrofa* chromosome localization). P-value, Bonferroni and FDR were obtained by a F-type test comparing the complete model (3) and the reduced model without interaction between gestational age and paternal genotype. (XLSX 73 KB)

Additional file 10: **Box-plot representation of the 10 tested genes in qPCR compared to their microarray expression '.pdf’ file.** **(A)** *ARG2*. **(B)** *PHKA1*. **(C)** *SLC38A4*. **(D)** *DLK1*. **(E)** *RASGRP3*. **(F)** *GPD1*. **(G)** *DUT*. **(H)** *GBP1*. **(I)** *IL1RAPL2*. **(J)** *SPG7*. All box-plots are normalized in log2. (PDF 71 KB)

Additional file 11: **Features of genes tested by real time RT-PCR '.xlsx’ file.** Gene names, description and primer (up and down) of the 10 genes tested by real time RT-PCR. (XLSX 13 KB)

## Abbreviations

BH: Benjamini-Hochberg
BIC: Bayesian information criterion
BP: Biological process
CC: Cellular component
CDK6: Cyclin-dependent kinase 6
CNV: Copy number variations
COL5A2: Collagen alpha-2(V) chain
CREM: cAMP responsive element modulator
DCI or ECI1: Enoyl-CoA delta Isomerase 1
DEP: Differentially expressed probes
FDR: False discovery rate
iid: independent and identically distributed
GO: Gene ontology
IGF2: Insulin-like growth factor 2
LDHA: Lactate dehydrogenase A
LW: Large white
MAGEL2: MAGE-like 2
MF: Molecular function
MS: Meishan
MYH3: Embryonic myosin heavy chain
NUSAP1: Nucleolar and spindle associated protein 1
OAA: Oxaloacetate
PCA: Principal component analysis
PC: Principal component
PGK1: Phosphoglycerate kinase 2
PCK2 or PEPCK: Phosphoenolpyruvate carboxykinase 2
PEP: Phosphoenolpyvruate
PYGM: Glycogen phosphorylase
qRT-PCR: Quantitative real time polymerase chain reaction
QTL: Quantitative trait locus
SGOL1: Shugoshin-like 1
SORD: Sorbitol dehydrogenase
STA: Specific target amplifications
STMN1: Stathmin 1.
