# Supplementary material for: Muscle transcriptomic investigation of late fetal development identifies candidate genes for piglet maturity
Source: BMC Genomics. 2014 Sep 17;15(1):797. doi: 10.1186/1471-2164-15-797 (PMC4287105; doi:10.1186/1471-2164-15-797)
Supplement: Supplementary file 5 — Additional file 5: Frequency distribution of Pearson’s correlation network '.pdf’ file. Frequency distribution of Pearson’s correlation between the entire set of 1516 genes (annotated or not) used to build our network. (PDF 36 KB) [file 12864_2014_6780_MOESM5_ESM.pdf]

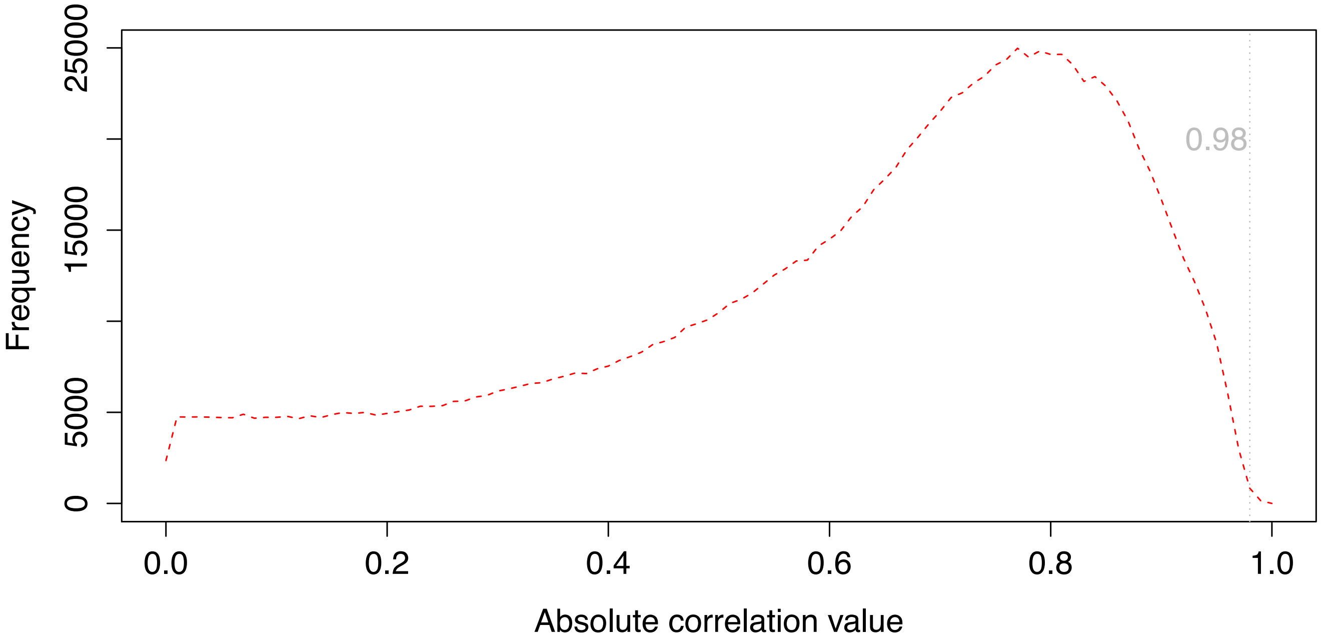


**Additional file 5:** Frequency distribution of absolute Pearson's correlation between the entire set of 1,516 genes (annotated or unannotated) used to build the relevance network.
