# Supplementary material for: Muscle transcriptomic investigation of late fetal development identifies candidate genes for piglet maturity
Source: BMC Genomics. 2014 Sep 17;15(1):797. doi: 10.1186/1471-2164-15-797 (PMC4287105; doi:10.1186/1471-2164-15-797)
Supplement: Supplementary file 10 — Additional file 10: Box-plot representation of the 10 tested genes in qPCR compared to their microarray expression '.pdf’ file. (A) ARG2. (B) PHKA1. (C) SLC38A4. (D) DLK1. (E) RASGRP3. (F) GPD1. (G) DUT. (H) GBP1. (I) IL1RAPL2. (J) SPG7. All box-plots are normalized in log2. (PDF 71 KB) [file 12864_2014_6780_MOESM10_ESM.pdf]

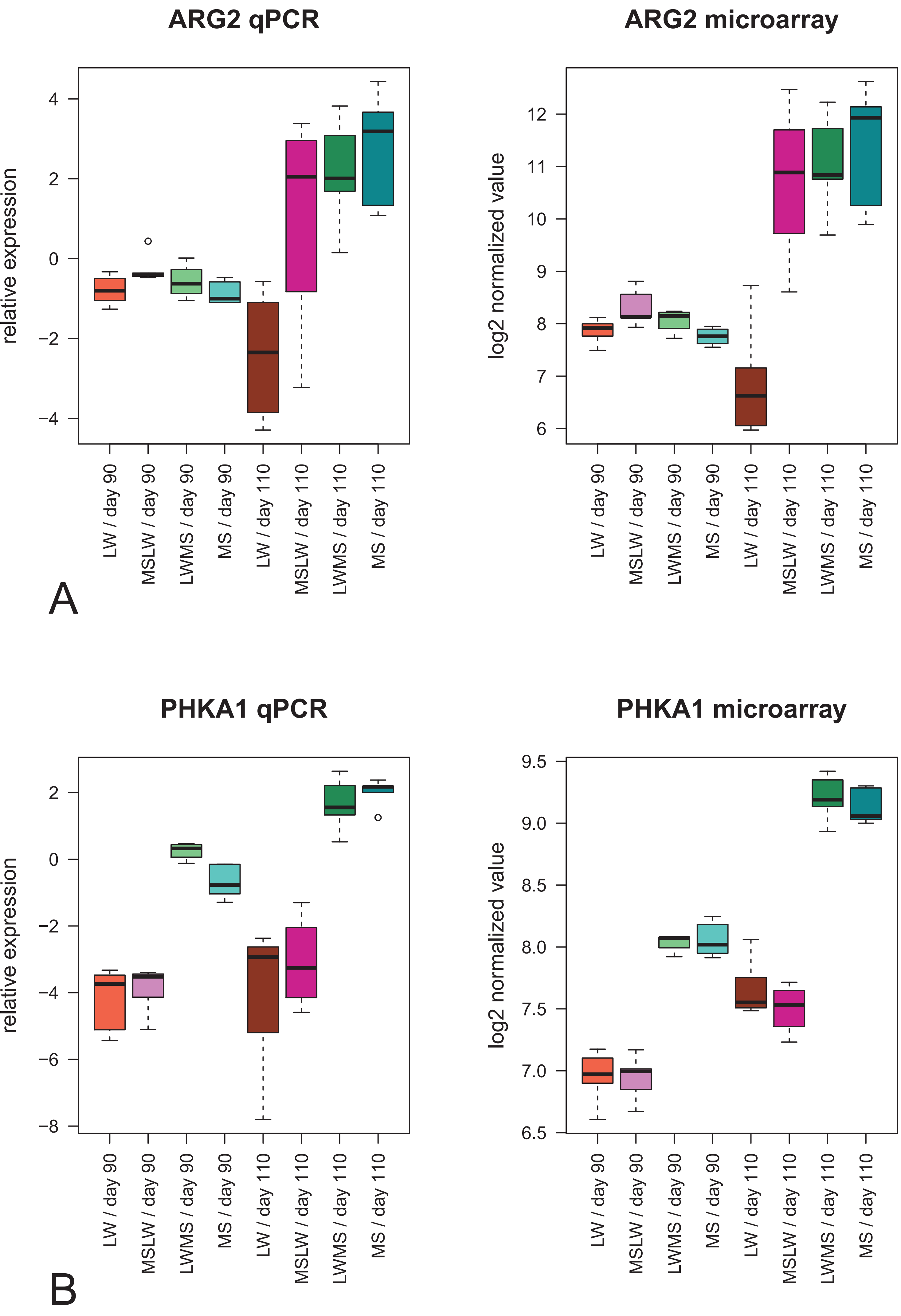

ARG2 qPCR
ARG2 microarray
relative expression
log2 normalized value
LW / day 90
MSLW / day 90
LWMS / day 90
MS / day 90
LW / day 110
MSLW / day 110
LWMS / day 110
MS / day 110
A
PHKA1 qPCR
PHKA1 microarray
B

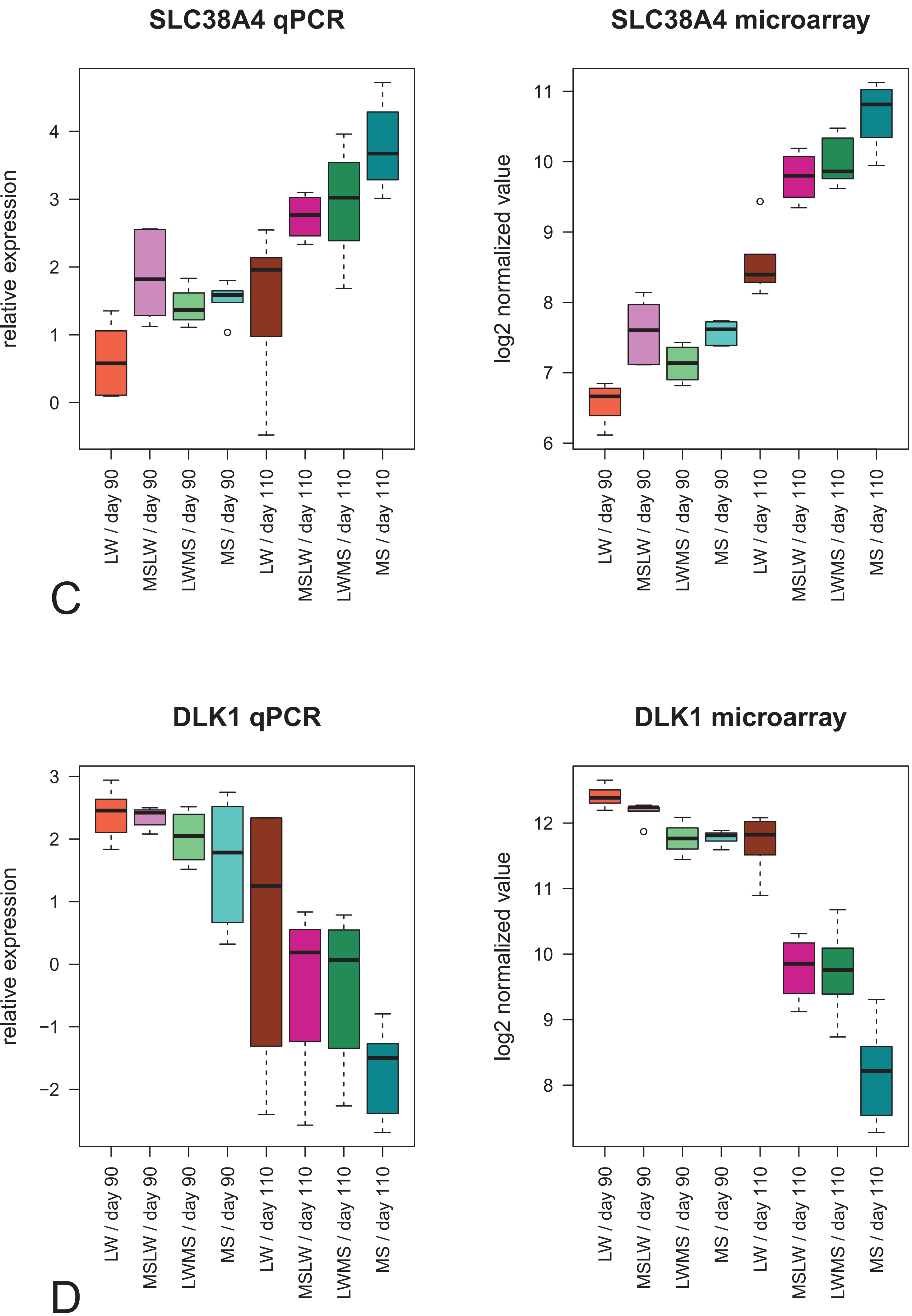
SLC38A4 qPCR
relative expression
0
1
2
3
4
SLC38A4 microarray
log2 normalized value
6
7
8
9
10
11
LW / day 90
MSLW / day 90
LWMS / day 90
MS / day 90
LW / day 110
MSLW / day 110
LWMS / day 110
MS / day 110
C
DLK1 qPCR
relative expression
−2
−1
0
1
2
3
DLK1 microarray
log2 normalized value
8
9
10
11
12
LW / day 90
MSLW / day 90
LWMS / day 90
MS / day 90
LW / day 110
MSLW / day 110
LWMS / day 110
MS / day 110
D

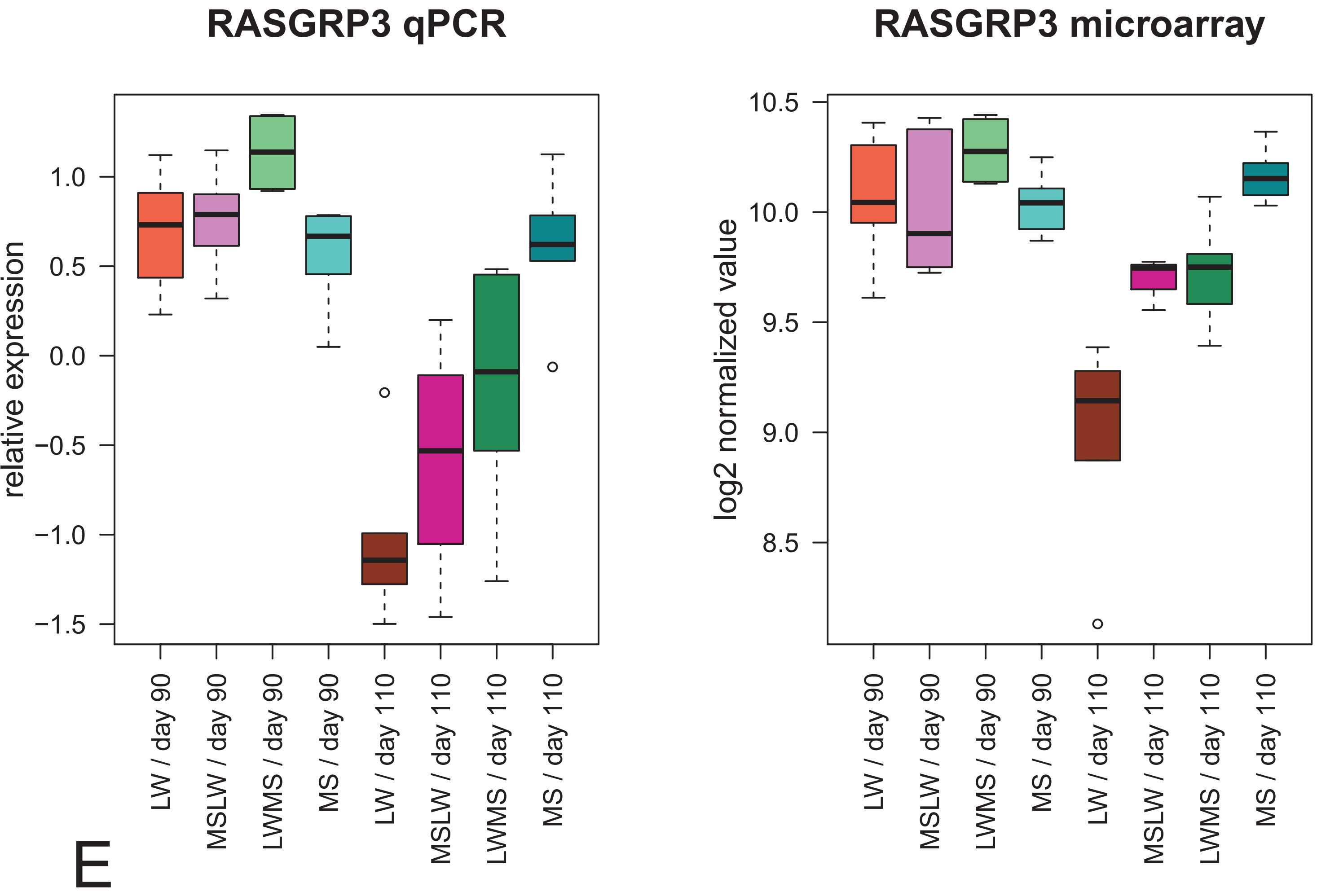
RASGRP3 qPCR
relative expression
1.0
0.5
0.0
−0.5
−1.0
−1.5
RASGRP3 microarray
log2 normalized value
10.5
10.0
9.5
9.0
8.5
LW / day 90
MSLW / day 90
LWMS / day 90
MS / day 90
LW / day 110
MSLW / day 110
LWMS / day 110
MS / day 110
E

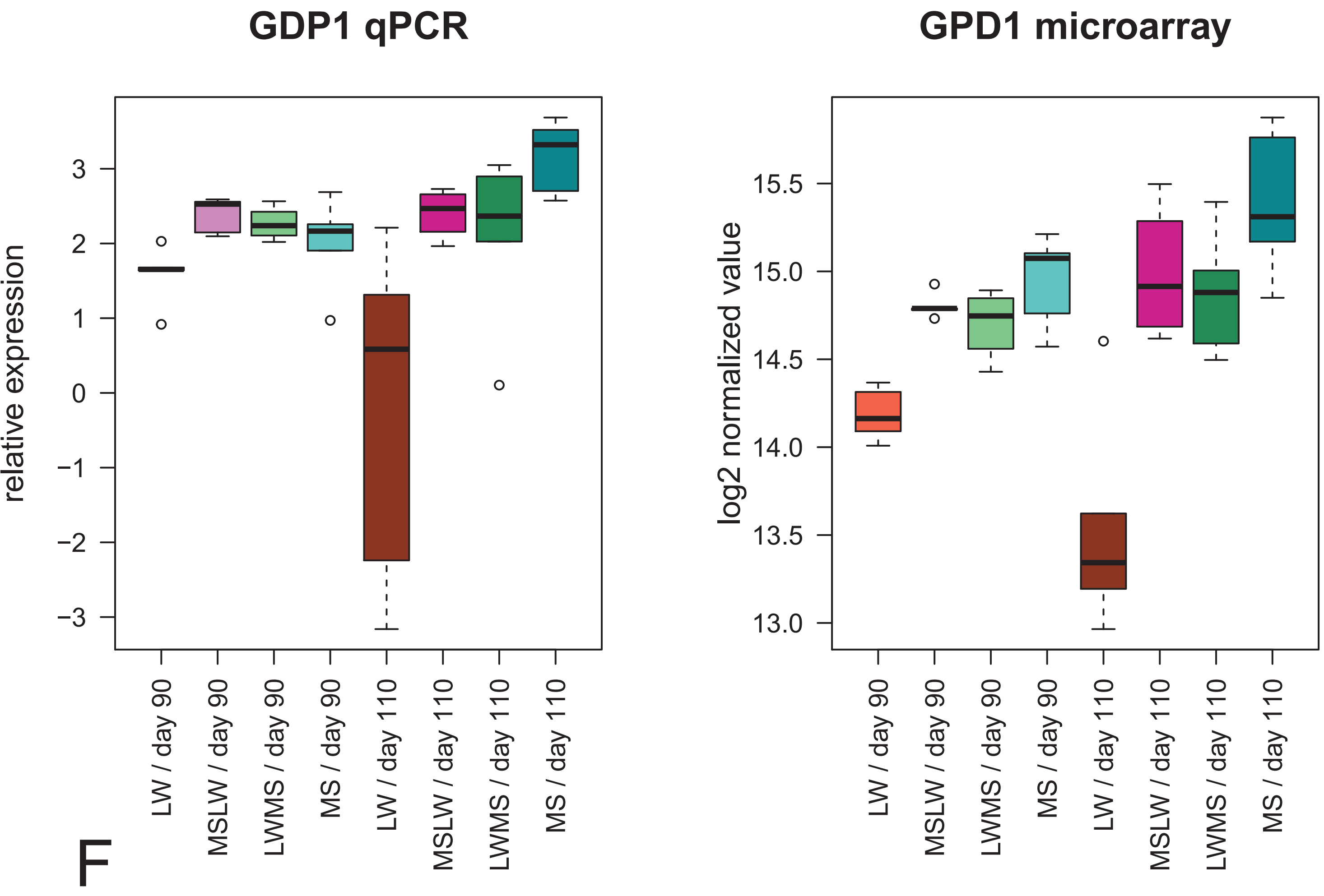
GDP1 qPCR
relative expression
3
2
1
0
−1
−2
−3
GPD1 microarray
log2 normalized value
15.5
15.0
14.5
14.0
13.5
13.0
LW / day 90
MSLW / day 90
LWMS / day 90
MS / day 90
LW / day 110
MSLW / day 110
LWMS / day 110
MS / day 110
F

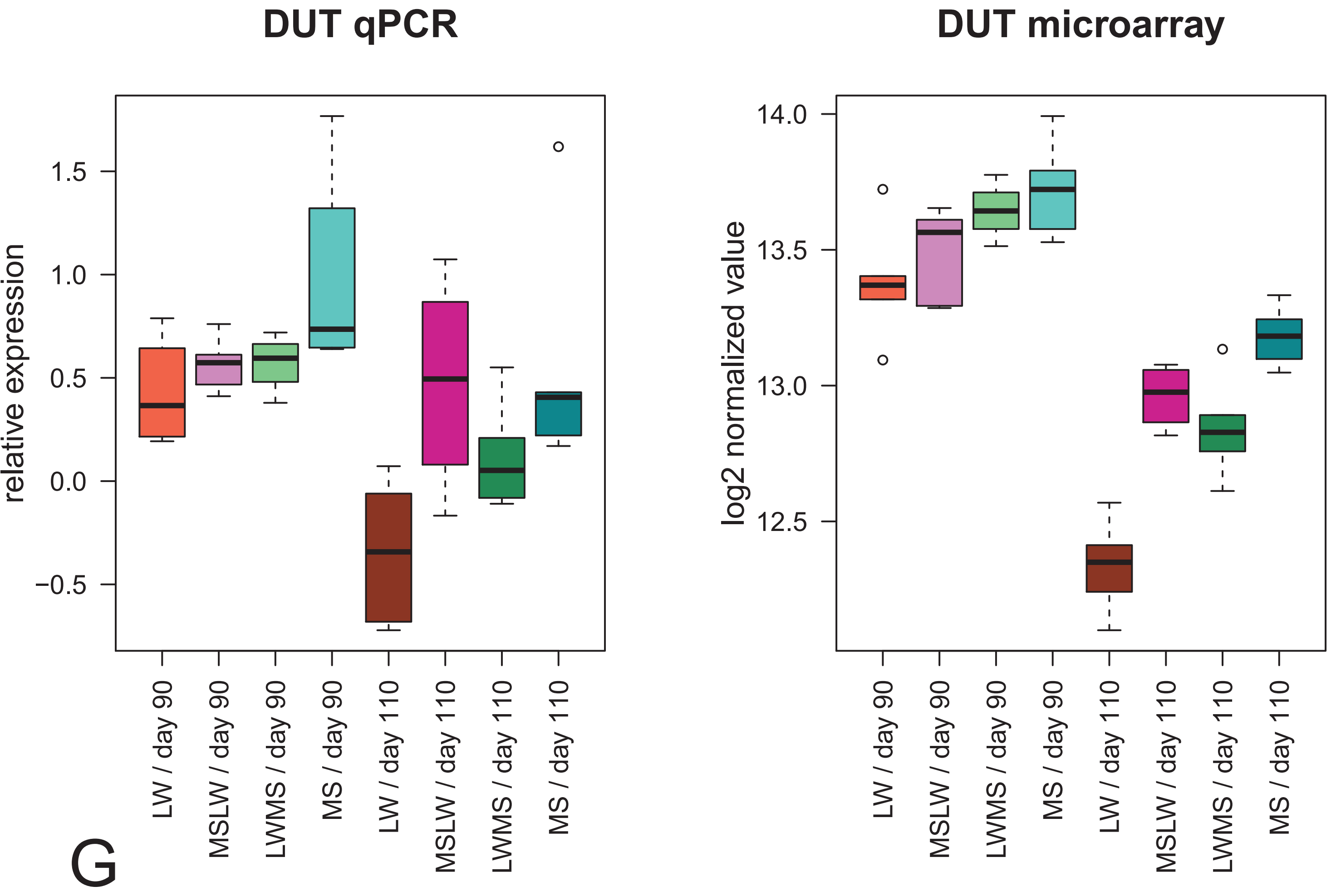
DUT qPCR
DUT microarray
relative expression
log2 normalized value
1.5
1.0
0.5
0.0
−0.5
14.0
13.5
13.0
12.5
LW / day 90
MSLW / day 90
LWMS / day 90
MS / day 90
LW / day 110
MSLW / day 110
LWMS / day 110
MS / day 110
G

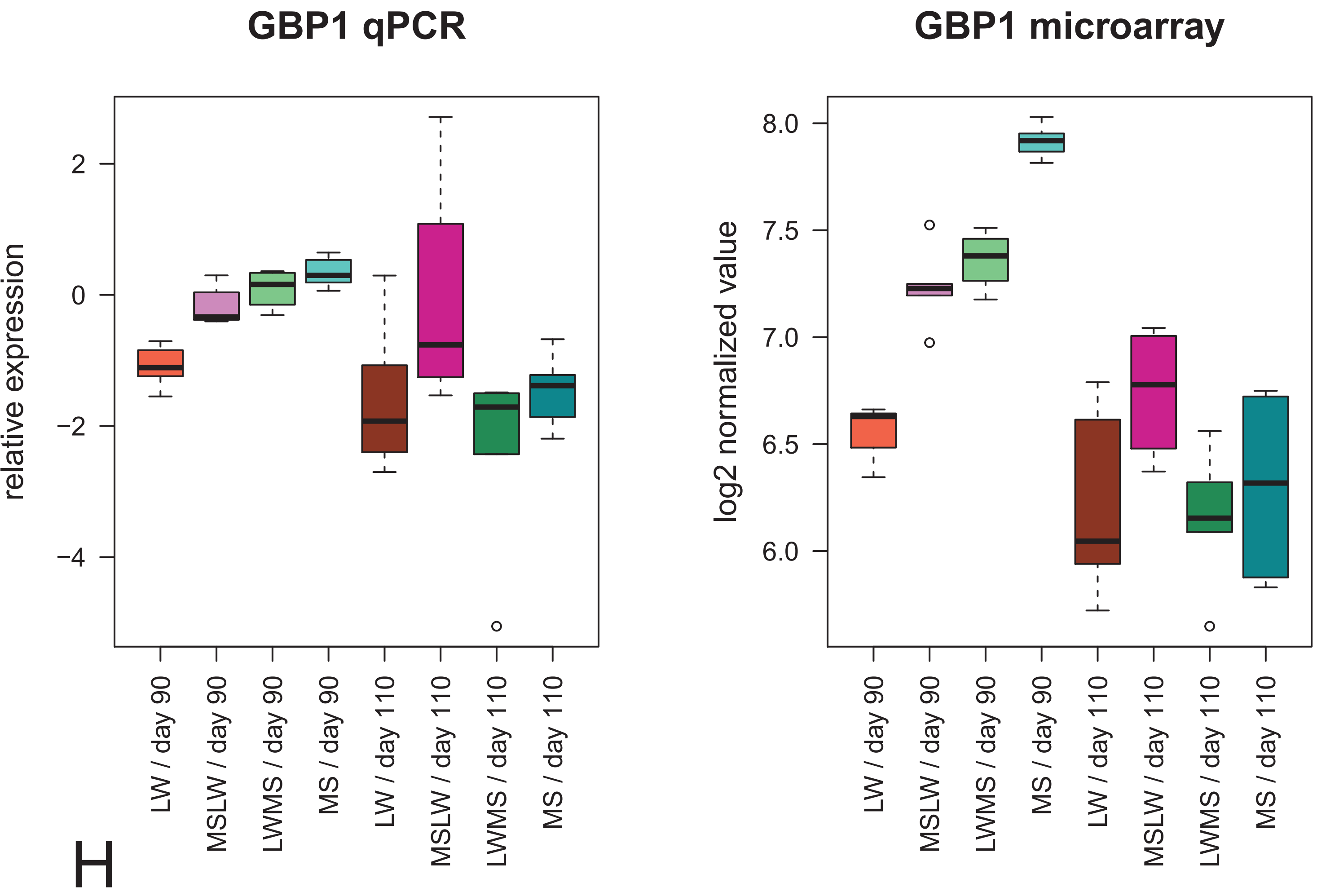
GBP1 qPCR
GBP1 microarray
relative expression
log2 normalized value
2
0
-2
-4
8.0
7.5
7.0
6.5
6.0
LW / day 90
MSLW / day 90
LWMS / day 90
MS / day 90
LW / day 110
MSLW / day 110
LWMS / day 110
MS / day 110
H

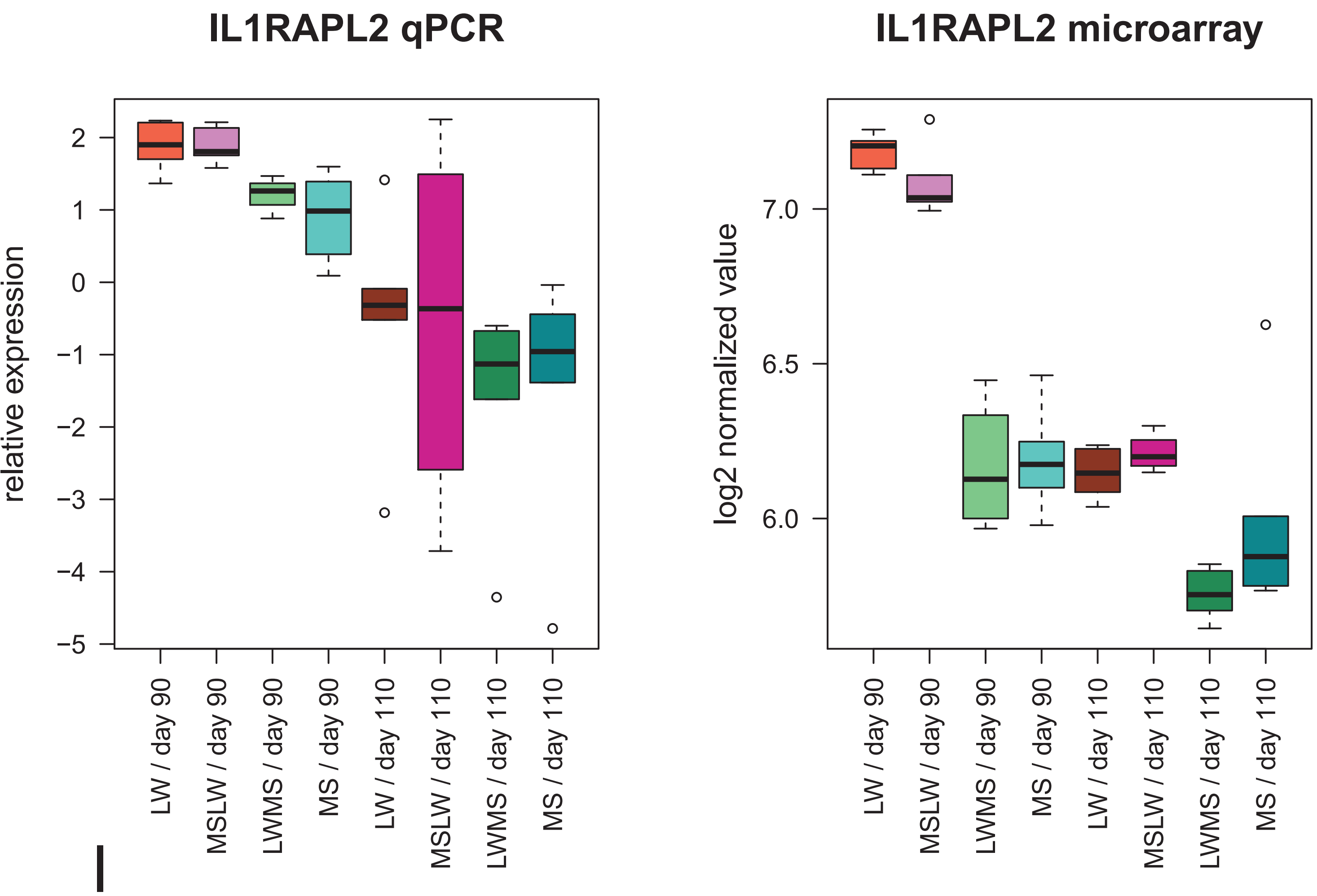
IL1RAPL2 qPCR
relative expression
2
1
0
−1
−2
−3
−4
−5
LW / day 90
MSLW / day 90
LWMS / day 90
MS / day 90
LW / day 110
MSLW / day 110
LWMS / day 110
MS / day 110
IL1RAPL2 microarray
log2 normalized value
7.0
6.5
6.0
LW / day 90
MSLW / day 90
LWMS / day 90
MS / day 90
LW / day 110
MSLW / day 110
LWMS / day 110
MS / day 110

I

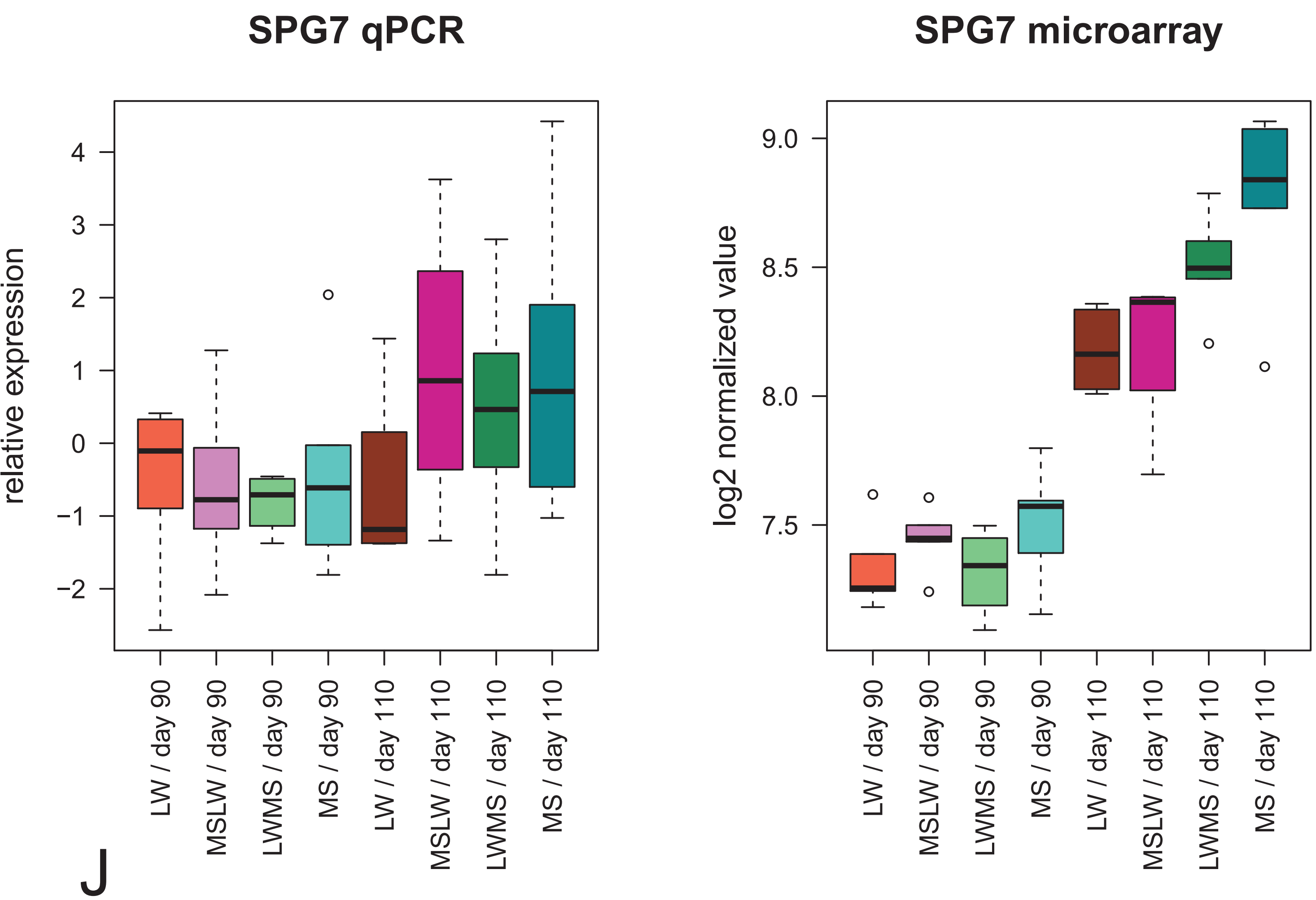
SPG7 qPCR
relative expression
4
3
2
1
0
−1
−2
LW / day 90
MSLW / day 90
LWMS / day 90
MS / day 90
LW / day 110
MSLW / day 110
LWMS / day 110
MS / day 110
SPG7 microarray
log2 normalized value
9.0
8.5
8.0
7.5
LW / day 90
MSLW / day 90
LWMS / day 90
MS / day 90
LW / day 110
MSLW / day 110
LWMS / day 110
MS / day 110

J
